# Highly logical and non-emotional decisions in both risky and social contexts: understanding decision making in autism spectrum disorder through computational modeling

**DOI:** 10.1007/s10339-024-01182-4

**Published:** 2024-03-25

**Authors:** Francisco Molins, Nour Ben-Hassen Jemni, Dolores Garrote-Petisco, Miguel Ángel Serrano

**Affiliations:** Department of Psychobiology, Universitat de València, Av. Blasco Ibáñez, 13, 46010 Valencia, Spain

**Keywords:** Autism spectrum disorder, Decision making, Framing effect, Ultimatum game, Computational modeling

## Abstract

In risky contexts, autism spectrum disorder (ASD) individuals exhibit more logical consistency and non-emotional decisions than do typical adults (TAs). This way of deciding could be also prevailing in social contexts, leading to maladaptive decisions. This evidence is scarce and inconsistent, and further research is needed. Recent developments in computational modeling allow analysis of decisional subcomponents that could provide valuable information to understand the decision-making and help address inconsistencies. Twenty-seven individuals with ASD and 25 TAs were submitted to a framing-task and the ultimatum game (UG). The Rescorla–Wagner computational model was used to analyze UG decisions. Results showed that in the UG, the ASD group exhibited a higher utilitarianism, characterized by lower aversion to unfairness and higher acceptance of offers. Moreover, this way of deciding was predicted by the higher economic rationality found in the framing task, where people with ASD did not manifest emotional biases such as framing effect. These results could suggest an atypical decision making, highly logical and non-emotional, as a robust feature of ASD.

## Introduction

The autism spectrum disorder (ASD) is a heterogeneous neurodevelopmental condition characterized by nuclear alterations in reciprocity, communication, and social interaction, as well as a rigid behavioral pattern, linked to deficits in social skills and difficulties in regulating emotions (American Psychiatric Association 2013; Jin et al. 2020; Reyes et al. 2019). Autobiographical and clinical reports also reveal difficulties in decision making throughout many situations and daily routines, manifesting mental “freezing” and exhaustion due to their highly logical and slow processing (Fujino et al. 2017; Luke et al. 2012). In fact, it has been recently suggested that atypical decision making is a robust feature of ASD (Shah et al. 2016). Understanding the decisional process of people with ASD could allow the identification of treatment strategies that favor their independence and quality of life.

Brosnan et al. (2016) highlighted that people with ASD manifest an excessively deliberative and logical reasoning, as well as little use of intuition. According to dual-process approaches (Brosnan et al. 2016; Evans 2008), this could lead to less economically irrational decisions (Rozenkrantz et al. 2021), especially in risky choices: simplified contexts where all decision’s alternatives and outcome’s probabilities are known (Volz & Gigerenzer 2012). In fact, this is what has been seen when studying framing effect (FE), the phenomenon whereby changes in the way alternatives are presented affect relative desirability of these alternatives (De Martino et al. 2006).

In this sense, typical adults (TAs) tend to prefer sure options in positive frames and risky options in mathematically identical, but negative frames (Manzoor et al. 2021). According to the classical economic model (Camerer 2003; von Neumann and Morgenstern 1944), FE is considered an irrational behavior since it breaks with one of the fundamental axioms of the rational decision making: the “invariance,” i.e., the logical consistency across decisions regardless of the frame (De Martino et al. 2006; Tversky and Kahneman 1989). In addition, it has been proposed that this phenomenon has an emotional origin, as a consequence of the disproportionate negative impact that potential losses produce in comparison with the positive impact of proportional gains (loss aversion; Sokol-Hessner and Rutledge 2019). Both FE and loss aversion neural bases are composed by structures such as the amygdala, insula or striatum (Christopoulos et al. 2009; Molins and Serrano 2019), regions that constitute fundamental nodes in the limbic system, which in turn is key for the production of emotional responses (Dalgleish 2004; Klumpp et al. 2012; Ledoux 2000; Phelps 2006; Thayer and Lane 2009). In fact, those patients with lesions in these regions, along with many other deficits in emotional processing and expression, also exhibit fewer of these biases (Bechara et al. 2003; Bechara and Damasio 2005; Clark et al. 2008). Additionally, genes that condition the expression of FE and loss aversion are directly related to the serotoninergic and dopaminergic pathways functioning, which in turn are intimately linked to emotion expression and regulation (Molins et al. 2022). Furthermore, various studies show that individuals who are better at regulating their emotions do not express as much FE or loss aversion during decision making (Sokol-Hessner et al. 2009, 2013). And the same holds true for those individuals who, due to their inability to accurately identify their emotions, i.e., those with high levels of alexithymia (Kinnaird et al. 2019; Shah et al. 2016), also exhibit fewer emotional biases when making decisions (Shah et al. 2016). This is why the occurrence of the FE during decision making is interpreted as synonymous with a greater impact of emotions on the decision-making process (Shah et al. 2016), as the aversion to losses would be conditioning decisions to a greater extent and hindering the use of logical and calculated strategies.

In ASD population, FE is significantly smaller (De Martino et al. 2008; Shah et al. 2016). These results suggest that people with ASD could be less influenced by the emotional response to losses during the decisional process, being able to isolate the objective value of alternatives and facilitating a logical, rule-based or utility-maximizing decisional strategy (Fujino et al. 2020; Shah et al. 2016). What is not known is whether this is because they exhibit a lower emotional reactivity to negative stimuli, or if the emotional response has the same intensity as in TAs but is not implemented in the decision-making process, as it seems to occur in those patients whose amygdala responds strongly to negative stimuli but fails to influence decisions due to deficits in the ventromedial prefrontal cortex (vmPFC) or the connectivity between these regions (Clark et al. 2008; Gu et al. 2015; Molins and Serrano 2019; Rolls et al. 2020). Though more rational in risky contexts, this way of deciding in individuals with ASD may be maladaptive if it is also maintained in other situations where emotions are particularly relevant (De Martino et al. 2008), as the case of social interaction proposed in the ultimatum game (UG).

The UG is a widely used tool to study social decision making (Hinterbuchinger et al. 2018). In this paradigm, the “proposer” must divide a hypothetical amount of money (e.g., 20€) between him/herself and the “responder.” The sharing can range from completely balanced (e.g., 10€ each) to completely unequal (e.g., keep 19€ and give 1€ to the responder). Then, the responder must decide whether to accept the split or, on the contrary, reject the offer and no player receive any money. Neuroeconomics research has revealed that human beings usually do not choose in a purely rational and utility-maximizing manner. Again, based on the classical economic model (Camerer 2003) proposers should always make the smallest possible offers, and responders should accept any offer greater than zero since this is the logical way to maximize benefits. But social decisions are the result of both, rational considerations, and emotional processes (Hinterbuchinger et al. 2018). So, across dozens of studies, typical proposers show generosity and a general preference for fairness and equality, consistently offering 40–45% of the stake in the UG (Hartley and Fisher 2018). Likewise, responders use to reject offers of less than a third since they feel aversion against inequity and prefer to punish selfish behavior to force more equitable offers in future interactions (Hinterbuchinger et al. 2018). This way of deciding is considered adaptive since it diminishes the impact of self-interests while it promotes cooperation and social cohesion (Hoffman et al. 2008).

Back to the ASD, it would be logical to think that their excessively economically rational way of deciding in risky contexts may also be manifested in the UG, making them fit the assumptions of the classical economic model. Some previous works point in this direction. So, it has been found that people with ASD distribute lower amounts when they make offers (Hinterbuchinger et al. 2018), and in turn, accept more offers, no matter how unfair (Hartley and Fisher 2018; Hinterbuchinger et al. 2018). Yet, these results are scarce and sometimes incongruent, as also were reported no differences between TAs and people with ASD when accepting offers (Trovato 2019), or even more altruistic behavior in the latter population when proposing the splits (Ikuse et al. 2018). These inconsistencies could be due to the fact that, as indicated by Gu et al. (2015), decisions in UG are usually analyzed in a general way, for example, by counting the total of accepted offers. However, decision making is not a single entity, but rather involves multiple subcomponents (Alacreu-Crespo et al. 2020). Thus, identifying the different cognitive and emotional subprocesses involved in the UG might show subtle alterations that are not captured by the traditional task scoring.

Recently, a computational model for the UG (Gu et al. 2015), in its responder version, has been developed. This model analyzes the underlying learning structure during the task. It assumes that responders have an internal norm on what is the fair amount that should be offered and identifies how sensitive the responder is to the breaking of this expectation, that is, how averse he/she is to inequity. As Gu et al. (2015) highlighted, the lower aversion to inequity should lead to a higher acceptance of offers. Complementarily, the model also analyzes whether the internal norm is persistent or is modified throughout the task, providing information on the adaptation to the changing context. Considering the less emotional decision making exhibited by the ASD population in risky contexts (Shah et al. 2016), it would be also expected to find less emotionality in the UG, i.e., that they will express a lower aversion to inequity. Moreover, given their rigid behavioral pattern (American Psychiatric Association 2013) it would also be logical to find a lower adaptation in their internal norm. As far as we know, this model has not been tested in ASD to date. Thus, our aim is to fill this gap and shed light on their decision-making process.

We hypothesize that, regarding TAs, the ASD population will exhibit the most economically rational way of deciding in both risky and social contexts. That is, they will show a lower FE in a risky-choice task, as well as a higher offers’ acceptance and a lower aversion to inequity in the UG. Moreover, they will show a lower internal norm adaptation in the latter task. Finally, although both risky and social contexts have been addressed separately in ASD, and their relationship has been theorized (e.g., Shah et al. 2016), to our knowledge, this association has never been directly tested. We expect that the way of deciding in risky contexts will predict decision making in the UG.

## Material and method

### Participants

Based on the effect size found in previous works on the ASD and both, the FE (Shah et al. 2016) and the UG (Hartley and Fisher 2018; Ikuse et al. 2018), an a priori power analysis using G*Power indicated a requisite between 21 (*η*^2^_*p*_ = 0.44, power = 80%, *α* = 0.05) and 36 (*η*^2^_*p*_ = 0.25, power = 80%, *α* = 0.05) participants per group to perform a general lineal model studying differences between people with ASD and TAs in FE and UG. We recruited 27 participants per group, but two participants had to be eliminated from the TAs group due to registration problems. So, our sample was finally composed by a total of 52 participants. The ASD group (*N* = 27; age: *M* = 32.19, *SD* = 10.44; women: *N* = 13, 48.1%) was recruited from a psychology center specialized in autism spectrum disorder. All members had a clinical diagnosis from an independent clinician according to DSM-5 criteria. TAs (*N* = 25; age: *M* = 27.56, *SD* = 10.66; women: *N* = 19, 76%) were recruited by the mean of non-probabilistic sampling method. All of them fulfilled the exclusion criteria as follows: not having physical, neurological, or psychiatric diseases; not consuming 10 or more cigarettes a day; not consuming drugs on regular bases; not having consumed drugs 24h before and not having taken stimulant drinks in the 2h before the assessment.

### Procedure

All participants signed informed consent before starting the session and were informed about which activities they had to perform, also insisting on that they were free to withdrawal their consent at any point of the study. Then, a sociodemographic questionnaire was administered asking about age, sex, socioeconomic status, as well as the inclusion criteria mentioned above. Afterward, the UG and FE tasks were administered. To respect the security measures derived from the COVID-19 situation, the administration was carried out telematically through an online protocol which was conducted remotely through online invitations sent to participants, instructing the participants on the necessary conditions to ensure the standardization of the measures. The study was approved by the Ethics Research Committee of the University of Valencia in accordance with the ethical standards of the 1969 Declaration of Helsinki.

### Instruments

#### Ultimatum game (UG)

**Fig. 1:**
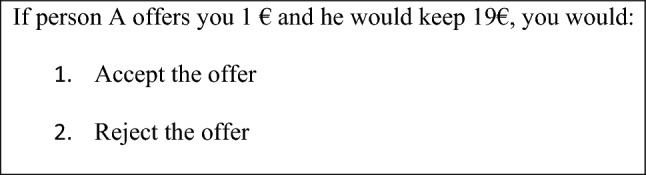
- Ultimatum game question example

Following the task from Gu et al. (2015), all participants played the role of the responder in the UG for a total of 45 trials. In each trial, participants were first offered a split of €20. Next, the subjects were presented with the choice options: accept or reject the offer. The offers were predetermined: 6 × €1, 6 × €2, 6 × €3, 6 × €4, 6 × €5, 3 × €6, 3 × €7, 3 × €8, 3 × €9, 3 × €10, presented in a randomized order by a hypothetical proposer (see Fig. 1). The total number of accepted offers was counted for each participant, allowing the average for each group to be extracted. In addition, the Rescorla–Wagner (delta) computational model (Gu et al. 2015) was applied to each participant to deepen in their underlying learning structure during the task.

##### Rescorla–Wagner (delta) Model

As was introduced, this model assumes that participants playing the UG have an internal norm (*f*_i_) on what is the fair amount that should be distributed to them. In addition, this norm can be updated as the context changes, i.e., the norm evolves as a function of observed offers (Xiang et al. 2013). Following Gu et al. (2015), the initial internal norm (*f*_0_) was fitted individually to each participant’s data (*f*_0_ ∈ [0,20]) and the Rescorla–Wagner rule (1972) was applied for updating the internal norm. For a detailed math description of the model, see Gu et al. (2015). Specifically, the model allowed to extract 3 parameters. α or “aversion to inequity” (α ∈ [0,1]) represents sensitivity to norm prediction error, in other words the individual aversion to unequal splits. The higher the α, the greater unwillingness to accept an offer below the internal norm (*f*_i_). ε or the “norm adaptation rate” (ε ∈ [0,1]) refers to how much the internal norm is modified according to the immediately preceding offer. A lower ε would indicate that the internal norm is more persistent. Finally, γ or the inverse temperature parameter (γ ∈ [0,1]) refers to the variability of the choices. The lower is γ, the lower consistence during the choices.

Each parameter of the model was estimated for each participant through hierarchical Bayesian analyses (HBA; see Ahn et al 2008 for more details), performed with the hBayesDM package (Ahn et al. 2017) for the R software. The hBayesDM uses Stan 2.1.1 (Stan Development Team 2017) with the Hamiltonian Monte Carlo (HMC) algorithm as MCMC for sampling the posterior distributions. Following Molins et al. (2021), we drew 40,000 samples, after burn-in of 23,333 samples, in three different chains (in sum, a total of 120,000 samples and 70,000 burn-in). The Gelman–Rubin test (Gelman & Rubin 1992) was used to study if the chains converged (*Ȓ*) to the target distribution. *Ȓ* values of all parameters were 1, which means that convergence was achieved. In addition, to confirm this convergence, the MCMC chains were visually inspected.

#### Framing effect (FE) task

**Fig. 2:**
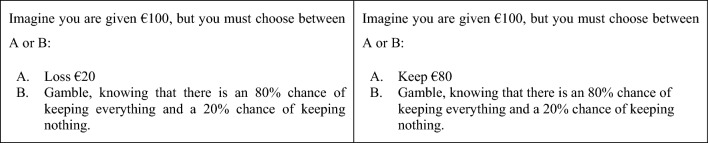
- Example of a FE trial in its two frames (negative vs. positive)

Participants completed an economical risky-choice framing task adapted from De Martino et al. (2006). They were informed that they received an amount of money (€25, €50, €75, and €100) and were asked to choose between a “sure” option and a “risky” option. On the one hand, the “sure” option could be presented either in a negative frame (e.g., “You lose €75”) or in a positive frame (“You keep €25”). It must be noted that the actual monetary value is equal in both options and the only difference is how they are worded. By the other side, the “risky” option consisted of gambling to either win or lose the whole amount of money announced before. The probabilities to win were 20%, 40%, 60%, and 80%, presenting the same number of trials for each percentage of riskiness. Types of frames, positive or negative, were randomly presented (16 loss and 16 gain frames), but for every positive trial, there was a complementary negative trial (see Fig. 2). Following the protocol from the original authors, participants were not given any feedback if they lost or won the gamble, in order to avoid a possible decisions shift because of the context dependence of risk preferences (Tversky & Kahneman 1992; Vermeer & Sanfey 2015; Xue et al. 2011). In addition, 16 “catch” trials were included, where one of the alternatives was notably beneficial in comparison with the other one (e.g., 95% chances to win/lose the whole amount vs. keeping/losing 50% of the initial amount). These were to assess the participant’s engagement and to assure that the answers were not random. Following the design developed by De Martino et al. (2006), any participant failing in more than 20% of these “catch” trials would be excluded from the study.

The FE was measured by comparing the preference (in percentage) to choose the “gamble” option over the “sure” option within each frame, in other words the difference between the percentages of trials the participant chose to gamble in a negative frame versus a positive frame.

### Statistical analyses

First, outliers were detected with the 2.5 standard deviations method and normality was checked through the Kolmogorov–Smirnoff test with the Lilliefors correction. The number of offers accepted in the UG, as well as each of the parameters extracted with the computational model, was compared between groups using ANOVAs. FE was analyzed through a repeated-measures ANOVA, comparing the percentage of gambles in positive vs negative frames, and including group as between-participants factor. After that, a two-stepped analysis was followed. First, a general linear model was conducted to study how FE and its interaction with the group predict UG results. Once the significant “framing x group” interaction was found, Pearson’s correlations by group were used to study the relation between variables. The *α* significance level was set at 0.05 and partial eta square (*η*^2^_*p*_) symbolizes the effect size. All analyses were performed with IBM SPSS Statistics 25, and the computational model was extracted with R i386.

### Community involvement statement

There was no community involvement in the reported study.

## Results

### Preliminary analyses

First, homogeneity between groups was tested. ASD and TAs groups did not show significant differences in age (ASD: *M* = 32.19, *SD* = 10.44; TAs: *M* = 27.56, *SD* = 10.66), *p* = 0.120; nor in socioeconomic status (ASD: *M* = 6.56, *SD* = 1.47; TAs: *M* = 5.88, *SD* = 1.25), *p* = 0.082. Nevertheless, the Chi-square test revealed that both groups included a different percentage of women (ASD: 48.1%; TAs: 76%) and men (ASD: 51.9%; TAs: 24%), *p* = 0.039, so the rest of the analyses were performed controlling for sex.

### Ultimatum game (UG)

As far as our first hypothesis is concerned, the ANOVAs carried out to study differences between groups in the UG revealed that TAs (*M* = 14.04, *SD* = 18.05) accepted on average fewer offers than the ASD group (*M* = 20.57, *SD* = 17.33), *F*(1, 50) = 4.96, *p* = 0.035, *η*^2^_*p*_ = 0.16. With respect to the parameters extracted with the computational model, the ASD group showed a lower aversion to inequity (α) and a higher norm adaptation rate (*ε*) than the TAs group; however, both groups did not differ in their consistence during choices or the inverse temperature parameter (γ) (see Table 1). In supplementary sensitivity analyses controlling for age, our results remained statistically consistent, reinforcing the robustness of our primary findings. To verify the assumptions of Gu et al. (2015), the relation between these parameters and the total accepted offers was studied. As expected, the greater the aversion to inequity (*r* = −0.836, *p* < 0.001) and the lower the norm adaptation rate (*r* = 0.598, *p* < 0.001), the fewer bets were accepted.

**Table 1:** | Differences between groups in the parameters of the Rescorla–Wagner (delta) model

	ASD (*N* = 27)	TAs (*N* = 25)	*F*	gl between	gl intra	*p*-value	*η*^2^_*p*_
*α*	*M* = 0.40 ± 0.27	*M* = 0.87 ± 0.12	7.40^**^	1	50	.01	.16
*ε*	*M* = 0.71 ± 0.11	*M* = 0.23 ± 0.13	17.50^***^	1	50	< .001	.81
*γ*	*M* = 0.37 ± 0.17	*M* = 0.29 ± 0.17	2.30	1	50	.13	.05

*ASD* autism spectrum disorder, *TAs* typical adults,* M* mean,  ±  *SD* standard deviation, *α* aversion to inequity, *ε* norm adaptation rate; γ, inverse temperature

**Significant contrast at the .01 level

***Significant contrast at the .001 level

### Framing Effect (FE)

**Fig. 3:**
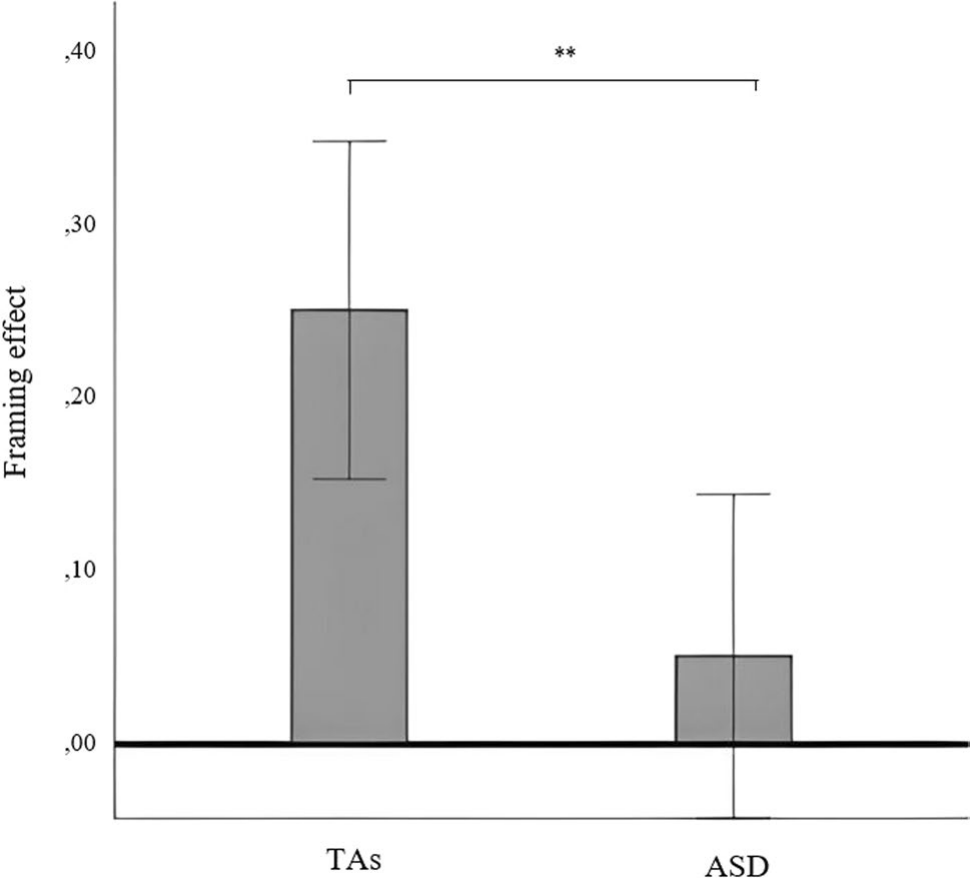
***–*** Difference between gambles accepted in negative vs positive frames (framing effect) by groups. TAs, *M* = 24.73%, *SD* = 29.82; ASD, *M* = 5%, *SD* = 14.32; **significant contrast at the .01 level

First, it was checked if any participant failed more than 20% of the “catch” trials. In our case, no participant was excluded for this reason. Then, a repeated-measures ANOVA including group as between-participants factor revealed the significant main effect of the frame (positive vs negative), *F*(1, 50) = 19.88, *p* < 0.001, η^2^_p_ = 0.30; and the significant frame x group interaction, *F*(1, 50) = 8.75, *p* = 0.005, η^2^_p_ = 0.16, on the percentage of gambles chosen. Analyzing these results in depth, the TAs group preferred to gamble on significantly more trials in the negative frame (*M* = 53.26%, *SD* = 35.19) than in the positive ones (*M* = 28.53%, *SD* = 26.83), *F*(1, 24) = 15.81, *p* = 0.001, *η*^2^_*p*_ = 0.41; however, no differences in gambling were shown between negative (*M* = 35.50%, *SD* = 28.16) and positive frames (*M* = 30.50%, *SD* = 24.29) in the ASD group, *F*(1, 26) = 3.04, *p* = 0.094, *η*^2^_*p*_ = 0.11. Thus, only the TAs group showed a significant FE. Complementarily, the intergroup ANOVA specifically conducted for the extracted FE variable (difference in the percentage of accepted bets in each context) reveals that the TA group (*M* = 24.73%, *SD* = 29.82) exhibited a significantly higher FE average compared to the ASD group (*M* = 5%, *SD* = 14.32), *F*(1, 50) = 8.75, *p* = 0.005, *η*^2^_*p*_ = 0.16 (see Fig. 3). Again, in further analyses incorporating age as a covariate, the statistical integrity of our principal results was consistently upheld.

### Relation between framing effect and Ultimatum game variables

A general linear model was conducted to study whether FE and its interaction with the group (ASD vs TAs) predict the UG variables. No significant results were found (*p*’s > 0.05). However, given that the ASD group did not show a significant FE, we repeated this analysis by splitting the FE variable into its two components: percentage of gambles in gain and loss frames. It was found that both the interaction "percentage of gambles in negative frames x group" (*B* = −5.49, *SE* = 1.79, *t* = −3.06, *p* = 0.004, *η*^2^_*p*_ = 0.19) and "percentage of gambles in positive frames × group" (*B* = −6.35, *SE* = 1.98, *t* = −3.21, *p* = 0.003, *η*^2^_*p*_ = 0.21) were negatively associated with the level of the α parameter. No other significant associations were found.

When these results were further explored through Pearson's correlations, no significant correlations were found between the framing variables and α parameter in the TAs group (*p*’s > 0.05); As for the ASD group, the α parameter was negatively correlated to the percentage of gambles in both the negative (*r* = −0.456, *p* = 0.05) and the positive frames (*r* = −0.485, *p* = 0.035). In sum, the higher the percentage of bets placed in both frames, the lower the aversion to unequal splits in UG.

## Discussion

The present study examined through computational modeling how people with ASD decide in social contexts. As expected, this population made more economically rational or utilitarian decisions than the TAs in the UG. So, they manifested a lower aversion to inequity and accepted a greater number of offers than TAs. Moreover, this way of deciding seems to be explained by the lack of emotional biases such as framing effect also shown in risky contexts. These results will be discussed below.

In line with some previous works (Hartley and Fisher 2018; Hinterbuchinger et al. 2018), the ASD group accepted significantly more offers than the TAs. Based on the classical economic model (Camerer 2003; von Neumann and Morgenstern 1944), since getting 1€ (while the proposer keeps 19€) has a higher utility than getting 0€, this would indicate that people with ASD were following an economically rational strategy and their decisions were not so guided by the feel of unfairness that emerge from selfish offers, as TAs usually do (Camerer 2003; Frith and Singer 2008; Hinterbuchinger et al. 2018). In fact, as the α parameter showed, the ASD group also exhibited a lower sensitivity to unfair splits, or lower aversion to inequity, than the TAs. Moreover, this level of aversion was negatively related to the number of accepted offers, supporting the weight that this emotional insensitivity would has on social decisions (Gu et al. 2015). Complementarily, we found that ASD and TAs groups did not differ in the consistency (γ) of their decisions, i.e., neither group made decisions more randomly than the other. However, and contrarily to our hypothesis, the ASD group showed a higher variability in his internal norm about fairness (ε) throughout the task. Yet, this might not necessarily be interpreted as greater flexibility and adaptation to the context, and this could also indicate greater volatility in his internal norm (Gu et al. 2015). In other words, greater inconsistency in the way offers are valued. Along with the lower sensitivity to unfairness, this could further evidence an atypical emotional processing in ASD, as many studies previously reported (e.g., Guastella et al. 2010; Teh et al. 2018; Wicker et al. 2008).

In fact, both a reduced aversion to inequity (*α*) and a highly variable internal norm about fairness (*ε*) have been associated with impairments in the ventromedial prefrontal cortex (vmPFC) (Gu et al. 2015). This region plays a key role in emotional processing and valuation (Gu et al. 2015; Rolls et al. 2020). So, patients with vmPFC lesions manifested decreased sensitivity to emotional cues, which leads to difficulties in reinforcement-learning (Bechara et al. 1994; Bechara & Damasio 2005), decreased guilty (Krajbich et al. 2009), decreased risk and loss aversion (Clark et al. 2008; Genauck et al. 2017; Shiv et al. 2005), and increased preference inconsistency in both risky (Fellows and Farah 2007) and social contexts (Gu et al. 2015). Multiple studies revealed, precisely, that people with ASD presented an abnormal functional brain development in vmPFC between childhood and adulthood (Murphy et al. 2017), and a smaller structure and function in this region in both children (Kishida et al. 2019; Swartz et al. 2013) and adults (Lau et al. 2020; Rolls et al. 2020; Salehinejad et al. 2021; Watanabe et al. 2012). In addition, these neural findings in ASD were also associated with poor decision making (Murphy et al. 2017) and social judgements (Watanabe et al. 2012). All this evidence would be in line with the economically rational (or non-emotional) way of deciding exhibited by the ASD group during the UG.

Moreover, our results support a common pattern between risky and social decisions in the ASD. Thus, as previously reported (De Martino et al. 2008; Shah et al. 2016), and as we hypothesized, the ASD group showed less emotional biases than the TAs group in a framing task. In fact, even if the ASD group appear to gamble more on negative frames, FE was not shown since no significant differences were observed between the percentage of bets placed on positive and negative frames. Moreover, this economically rational way of deciding found in the ASD group was associated with their utilitarianism in social contexts. So, the more bets they accepted in both frames, i.e., the less framing effect and loss aversion (Kahneman 2003; Tversky and Kahneman 1989), the less inequity aversion they expressed during the UG. One possible interpretation of our results would be that individuals with ASD could be ignoring emotional cues, such as the negative impact that losses typically produce or the aversion generated by unfair economic distributions, and guiding their decisions through an entirely economically rational strategy (Camerer 2003; Rozenkrantz et al. 2021), in both risky and social contexts. Therefore, an atypical emotional processing may be a central point in the ASD decision making, regardless of the context. Nevertheless, as suggested Kinnaird et al. (2019), rather than a core feature of ASD, emotional processing difficulties could also reflect co-occurring alexithymia. This is a personality trait, heightened in ASD compared to the general population, and characterized by difficulties identifying and describing one’s own emotions (Kinnaird et al. 2019; Shah et al. 2016). In addition, alexithymia seems to be behind a less emotional risky decision making in individuals without ASD (Manzoor et al. 2021), although not in the ASD population (Shah et al. 2016). Thus, it is necessary to clarify whether the economically rational way of deciding found in ASD is due to the disorder itself or, on the contrary, is caused by other factors such as alexithymia.

One of the limitations of this study is the absence of complementary emotional measures such as alexithymia, emotional regulation capacity, as well as other physiological or neural correlates that could shed additional light on the emotions influence on the decisional process. Moreover, although sex has been controlled for in the analyses, the disproportionate sample does not allow us to explore what variance explains this factor. Future research is needed given that differences have been found in the capacity to process and regulate emotions between men and women (Rattel et al. 2020). Furthermore, we also suggest for further studies to measure more auxiliary variables that might be interfering such as education level or culture, as well as to include larger sample size as it adds more robustness to the results. By the other side, since this study only addressed the role of the responder as the Rescorla–Wagner (delta) computational model can only be applied to this modality (Gu et al. 2015), it would be important to further study whether these emotional deficits also impact on the proposer role, for example, manifesting greater selfishness. Finally, it would be relevant to consider in the future whether the results may vary when real money is at stake, as many studies are conducted with hypothetical money, but this mode could dampen emotional responses and impact the outcomes.

Nevertheless, this is the first study connecting risky and social decision making in ASD. Moreover, it explored through computational modeling the underlying cognitive process in the latter context. Results are consistent with previous research and point to a lower emotional sensitivity during the decisional process, impeding emotional cues from guiding decisions and, therefore, having to rely on an extremely rational strategy (Rozenkrantz et al. 2021). In line with the need to contextualize posed by the ecological rationality approach (Brighton and Gigerenzer 2012), while this way of deciding could be useful in risky contexts, it could be responsible for maladaptation in social contexts. All these results reinforce the idea that atypical decision making is a robust feature of ASD and open up possible therapeutic targets, addressing ASD through techniques that improve emotional awareness and regulation, or even, as some recent studies point out (Salehinejad et al. 2021), by enhancing vmPFC activity through transcranial direct current stimulation.
